# Repply to: Knowledge and practices of postmenopausal women regarding vitamin D and osteoporosis

**DOI:** 10.61622/rbgo/2026rbgo108L24R

**Published:** 2026-10-13

**Authors:** Yasmin Bastos-Silva, Luiza Borges Aguiar, Luiz Francisco Baccaro, Adriana Orcesi Pedro, Lúcia Costa-Paiva

**Affiliations:** 1 Universidade Estadual de Campinas Faculdade de Ciências Médicas Departmento de Tocoginecologia Campinas SP Brazil Departmento de Tocoginecologia, Faculdade de Ciências Médicas, Universidade Estadual de Campinas, Campinas, SP, Brazil

Dear Editor,

We would like to thank the authors for their thoughtful comments on our article, *"Knowledge and practices of postmenopausal women regarding vitamin D and osteoporosis."* We appreciate the opportunity to further discuss some of the findings and implications of our study. We agree that the discrepancy between the relatively high prevalence of vitamin D supplementation and the limited knowledge about vitamin D observed in our study is an important finding. Although 67.18% of the participants reported using vitamin D supplements, only 32.69% achieved four or more correct answers on the vitamin D knowledge questions. This finding suggests that supplementation does not necessarily imply adequate understanding of the role, sources, and potential benefits of vitamin D. As appropriately highlighted by the authors of the letter, this issue has also been reported in other populations. We also agree that health literacy and educational level may play an important role in the understanding of health-related information. In our study, higher educational attainment was strongly associated with greater knowledge about vitamin D. However, because of the cross-sectional design of our study, we cannot establish a causal relationship between educational level, health literacy, and vitamin D knowledge. Moreover, health literacy itself was not directly assessed. We therefore agree that future studies should specifically evaluate health literacy and investigate whether educational interventions can improve not only knowledge but also attitudes, practices, and adherence to preventive or therapeutic recommendations.

Regarding sun exposure and photoprotection, we agree that this is a particularly complex issue. In our study, 68.46% of women reported using sunscreen and 55.77% reported using other photoprotective measures, while 53.85% considered that they did not expose themselves sufficiently to sunlight. These findings illustrate the difficulty of balancing potential vitamin D synthesis with the well-established need to reduce excessive ultraviolet radiation exposure. Importantly, our study was not designed to determine whether the participants had adequate serum vitamin D concentrations or to establish an optimal pattern of sun exposure. We therefore agree with the authors that individualized counseling is important. Rather than encouraging prolonged or excessive sun exposure, health professionals should consider the individual characteristics and risk factors of each patient when discussing vitamin D and bone health. Dietary sources, supplementation when clinically indicated, and appropriate photoprotection should all be considered within a comprehensive approach to osteoporosis prevention.^(1,2)^

We also appreciate the authors’ emphasis on the role of specialized healthcare services and educational strategies. Our findings suggest that simply providing access to healthcare or prescribing supplementation may not be sufficient to ensure that women understand the importance of vitamin D and osteoporosis prevention. Educational interventions adapted to patients’ educational backgrounds and health literacy levels may represent an important strategy to improve preventive care.^(3)^

Finally, we agree that further research is needed to better understand the relationship between health literacy, education, knowledge, attitudes, and practices regarding vitamin D and osteoporosis. Prospective studies and intervention trials could help determine whether improving knowledge translates into meaningful changes in health behaviors and clinical outcomes. We thank the authors once again for their valuable contribution to the discussion and for highlighting important perspectives that complement the findings of our study.
